# Verletzung der A. profunda femoris mit transfusionspflichtiger Blutung durch sekundäre Dislokation des Trochanter minor nach Osteosynthese einer pertrochantären Femurfraktur

**DOI:** 10.1007/s00113-020-00854-y

**Published:** 2020-08-20

**Authors:** D. Hertig, R. Thalmann, B. Rufer

**Affiliations:** 1 Klinik für Orthopädie und Traumatologie, Sonnenhof Bern, Salvisbergstr. 4, 3006 Bern, Schweiz; 2 Stiftung Lindenhof, Campus SLB, Schweizerisches Institut für Translationale und Unternehmerische Medizin, Freiburgstrasse 3, 3010 Bern, Schweiz

**Keywords:** Blutung A. profunda femoris, Postoperative Anämie, Fragmentdislokation, Proximaler Femurnagel, Gefässembolisation, Bleeding of deep femoral artery, Postoperative anemia, Dislocation of bone fragment, Proximal femoral nailing, Embolization

## Abstract

Ein 95-jähriger Patient erlitt eine transfusionspflichtige Blutung nach Osteosynthese einer pertrochantären Femurfraktur mittels proximalem Femurnagel. Das bei der Erstmobilisation sekundär dislozierte Fragment des Trochanter minor perforierte einen Seitenast der A. profunda femoris. Die Blutung konnte mittels Embolisation gestillt werden, und der Patient wurde wenige Tage später mit stabilem Hämoglobinwert entlassen. Wiederholt sinkende Hämoglobinwerte trotz mehrmaliger Bluttransfusion weisen auf eine aktive Blutung hin. Die genaue Identifikation der Blutungsquelle nach Osteosynthese proximaler Femurfrakturen ist entscheidend für die korrekte Therapie.

## Abstract (en)

A 95-year-old patient suffered bleeding that required a transfusion after closed reduction and internal fixation (CRIF) of an intertrochanteric femoral fracture with a proximal femoral nail. After mobilization of the patient the secondarily dislocated fragment of the lesser trochanter perforated a side branch of the profunda femoris artery. The bleeding could be stopped by embolization and a few days later the patient could be discharged with stable hemoglobin levels. A continuing decrease of hemoglobin levels despite repeated transfusion of red blood cells is indicative of active bleeding. Accurate identification of the source of the bleeding after osteosynthesis of proximal femoral fractures is decisive for the correct treatment.

## Falldarstellung

### Anamnese

Die Zuweisung durch den Rettungsdienst erfolgte nach unbeobachtetem Sturz in der Alterswohnung mit immobilisierenden Hüftschmerzen links. Der Patient war zuvor selbstständig am Rollator mobil. Als einziges blutverdünnendes Medikament wurde Aspirin, 100 mg, einmal täglich eingenommen.

### Status und Befunde

Bei Spitaleintritt war der Patient hämodynamisch stabil. Klinisch zeigte sich eine Druckdolenz über dem linken Trochanter major mit immobilisierenden Schmerzen. Das Bein war nicht verkürzt oder außenrotiert mit intakter peripherer Sensomotorik. Die Fußpulse waren nicht palpabel, die Füße aber warm und das Hautkolorit normal.

### Diagnose

**Figure 1:**
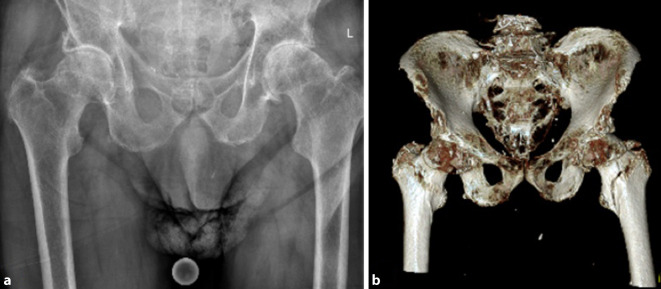

Konventionell radiologisch präsentierten sich im axialen Bild eine Konturunterbrechung zwischen Trochanter major und Trochanter minor sowie eine fortgeschrittene Koxarthrose links (Abb. 1a). Die Computertomographie zeigte eine nichtdislozierte pertrochantäre Femurfraktur links, 31 A1.3 nach AO-Klassifikation. Der Trochanter minor stand noch in Kontinuität mit dem Calcar bzw. dem Kopf‑/Schenkelhalsfragment. Die Frakturlinie lief distal des Trochanter minor in die Femurmetaphyse aus (Abb. 1b).

### Therapie und Verlauf

**Figure 2:**
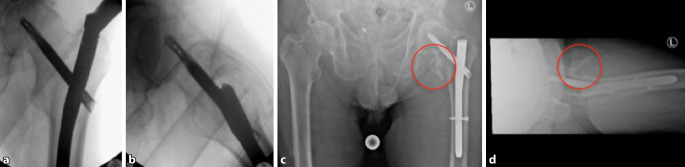

**Figure 3:**
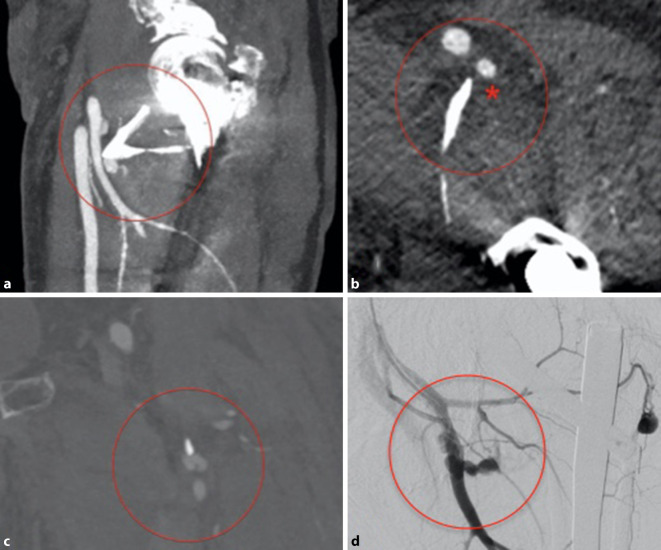

Die Osteosynthese der Fraktur wurde mit einem proximalen Femurnagel (TFNAdvanced, DePuy Synthes, Synthes GmbH, Eimattstrasse 3, 4436 Oberdorf, Switzerland) durchgeführt. Die postoperative Röntgenkontrolle mittels Bildverstärker zeigte anatomische Stellungsverhältnisse des proximalen Femurs, insbesondere des Trochanter minor (Abb. 2a, b). Der intraoperative Blutverlust betrug 600 ml, wobei auch das ausgeprägte Frakturhämatom abgesaugt wurde. Zur Thromboseprophylaxe wurden 6 h postoperativ 40 mg Enoxaparin subkutan verabreicht. Der Patient konnte am Folgetag mit einer Gehhilfe mobilisiert werden. Am zweiten postoperativen Tag äußerte der Patient zunehmende Schmerzen, und am linken Oberschenkel fiel eine ausgeprägte Schwellung auf. Im konventionellen Röntgenbild war neu eine Dislokation des Trochanter-minor-Fragments nach kranial zu erkennen (Abb. 2c, d), bei ansonsten weiterhin korrekter Stellung des proximalen Femurs und des Marknagels. Trotz Substitution von insgesamt 4 Erythrozytenkonzentraten (EK) sank der Hämoglobin(Hb)-Wert auf 7,5 g/dl bei einem präoperativen Ausgangswert von 13,3 g/dl. Zur Identifikation der Blutungsquelle wurde eine Kontrastmittelcomputertomographie veranlasst, die eine Blutung aus einem Seitenast der A. profunda femoris (APF) zeigte (Abb. 3a–c). Sehr wahrscheinlich wurde die Gefäßwand durch die Spitze des im Rahmen der Erstmobilisation sekundär dislozierten Trochanter-minor-Fragments perforiert. Von den interventionellen Radiologen wurde mittels Coils der betroffene Seitenast der APF embolisiert (Abb. 3d und 4).

**Figure 4:**
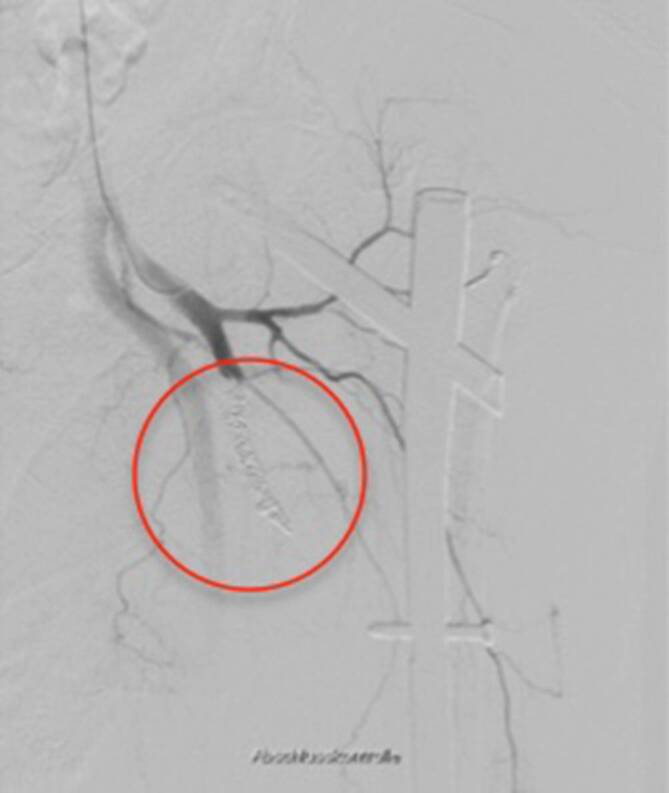

**Figure 5:**
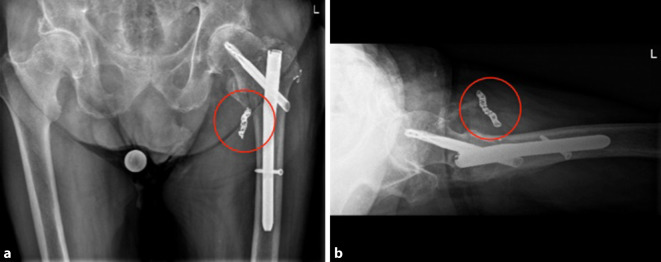

Im Anschluss stiegen die Hb-Werte sukzessive an. In der klinischen und radiologischen Verlaufskontrolle 6 Wochen postoperativ war der Patient am Rollator schmerzfrei mobil, mit warmer Peripherie und intakter Sensorik. Im Röntgenbild zeigte sich unverändert korrekt einliegendes Osteosynthesematerial (Abb. 5).

## Diskussion

Hüftgelenknahe Frakturen sind mit rund 20 % aller osteoporotischen Frakturen die häufigste Fraktur des älteren Menschen [2]. Allein in Deutschland mussten im Jahr 2014 über 100.000 Patienten mit proximaler Femurfraktur operiert werden [3]. Die Mortalität während des Spitalaufenthalts nach Osteosynthese einer proximalen Femurfraktur wird mit 1,7 % bis zu 2,9 % [4, 5] angegeben. Die Einjahresmortalität steigt sogar auf 10–30 % [6].

Die am häufigsten zur Stabilisierung dieser Frakturen eingesetzten Implantate sind dynamische Schrauben- und Plattensysteme sowie intramedulläre Nägel. Der Trochanter minor wird in der Regel nicht separat fixiert, aufgrund des Risikos iatrogener Verletzungen der Gefäße zur Durchblutung des Femurkopfes, die zu einer Femurkopfnekrose führen können. Eine Ausnahme stellen beispielsweise Frakturen mit Verlust des posteromedialen Supports am Calcar mit ausreichend großem Fragment des Trochanter minor dar, die distal des Trochanter minor eine Fixation mittels Cerclagen erlauben.

Durch die Operation verursachte lokale Komplikationen umfassen Wundheilungsstörungen, Hämatome und postoperative Wundinfektionen. Verletzungen der APF können beispielsweise durch das Trauma, iatrogen durch Fehlplatzierung von Kirschner-Drähten oder Retraktoren sowie beim Bohren oder durch überstehende Verriegelungsbolzen verursacht werden. Die sekundäre Dislokation eines Knochenfragments mit nachfolgender Gefäßverletzung ist sehr selten. Sie wird hauptsächlich in Fallberichten beschrieben [7, 8]. Barquet et al. gaben 2015 in einem Review eine Inzidenz von 0,49 % an. Die zwei häufigsten Formen sind Lazerationen und Pseudoaneurysmata [9, 10]. Die Symptomatik kann von lediglich atypischen inguinalen Schmerzen durch das paravasale Hämatom bis hin zum lebensbedrohlichen hypovolämischen Schock bei anämisierender Blutung gehen.

Potenza et al. berichten in einem dieser Kasuistik ähnlichen Fall über einen 81-jährigen Mann, der nach Osteosynthese einer pertrochantären Femurfraktur mittels proximalem Femurnagel ohne intraoperative Komplikationen hämodynamisch instabil wurde und im Schock rehospitalisiert werden musste. Auch hier zeigte sich im Kontrastmittel-CT eine Lazeration eines Astes der APF, verursacht durch die Spitze des sekundär dislozierten Trochanter minor [1].

Auch Pseudoaneurysmata können rupturieren und sollten daher gefäßchirurgisch oder endovaskulär versorgt werden. Weitere potenzielle Komplikationen der Pseudoaneurysmata sind die Kompression benachbarter neurovaskulärer Strukturen, Thromboembolien oder Infektionen [10].

Nachblutungen werden wahrscheinlich häufiger durch eine Verletzung der Vv. perforantes im Rahmen des Traumas oder der Operation durch den chirurgischen Zugang für die Schenkelhalsklinge/-schraube bzw. die distale Verriegelung verursacht. Doch ein Revisionseingriff ohne vorgängige Identifikation der Blutungsquelle kann einerseits die Blutung nicht stoppen (im Fall einer verpassten Läsion der APF) und stellt für den Patienten eine Belastung durch die erneute Narkose dar. Auch steigt das Infektionsrisiko durch das Eröffnen des Wundsitus.

In der hier dargestellten Kasuistik eines geriatrischen Patienten mit Osteosynthese einer proximalen Femurfraktur führte die sekundäre Dislokation des Trochanter minor zur Perforation eines Seitenastes der APF mit transfusionspflichtiger Blutung. Wiederholt sinkende Hb-Werte trotz Transfusion von EK deuten auf eine persistierende Blutung hin. Um die Blutungsquelle zu identifizieren, soll eine Abklärung mittels i.v.-Kontrastmittel-CT erfolgen. Die Therapie hängt von der Blutungsquelle ab und kann entweder offen durch einen chirurgischen Eingriff erfolgen, beispielsweise bei Verletzungen der Vv. perforantes. Arterielle Verletzungen von Seitenästen der APF können durch interventionelle Radiologen gecoilt werden.

## Fazit für die Praxis

- Nach Osteosynthese pertrochantärer Femurfrakturen empfehlen sich regelmäßige Hämoglobinkontrollen, um eine persistierende Blutung nicht zu verpassen.
- Bei Verdacht auf eine persistierende Blutung postoperativ hilft eine Kontrastmittelcomputertomographie, die Blutungsquelle zu identifizieren.
- Die Therapie unterscheidet sich in Abhängigkeit von der Blutungsquelle (offene chirurgische Revision vs. endovaskuläre Verfahren).
